# Gadolinium Chloride Attenuates Sepsis-Induced Pulmonary Apoptosis and Acute Lung Injury

**DOI:** 10.5402/2012/393481

**Published:** 2012-11-01

**Authors:** Osama A. Kishta, Peter Goldberg, Sabah N. A. Husain

**Affiliations:** Department of Critical Care, Medicine McGill University Health Centre and Meakins-Christie Laboratories, McGill University, Montreal, Quebec, Canada H3A 1A1

## Abstract

Gadolinium chloride (GdCl_3_), a Kupffer cells inhibitor, attenuates acute lung injury; however, the mechanisms behind this effect are not completely elucidated. We tested the hypothesis that GdCl_3_ acts through the inhibition of lung parenchymal cellular apoptosis. Two groups of rats were injected intraperitoneally with saline or *E. coli* lipopolysaccharide. In two additional groups, rats were injected with GdCl_3_ 24 hrs prior to saline or LPS administration. At 12 hrs, lung injury, inflammation, and apoptosis were studied. Lung water content, myeloperoxidase activity, pulmonary apoptosis and mRNA levels of interleukin-1**β**, -2, -5, -6, -10 and TNF-**α** rose significantly in LPS-injected animals. Pretreatment with GdCl_3_ significantly reduced LPS-induced elevation of pulmonary water content, myeloperoxidase activity, cleaved caspase-3 intensity, and attenuated pulmonary TUNEL-positive cells. GdCl_3_ pre-treatment upregulated IL-1**β**, -2 and -10 pulmonary gene expression without significantly affecting the others. These results suggest that GdCl_3_ attenuates acute lung injury through its effects on pulmonary parenchymal apoptosis.

## 1. Introduction

Acute lung injury (ALI)/acute respiratory distress syndrome (ARDS) is a frequent complication of sepsis affecting approximately from 25 to 40% of septic patients and carries a mortality of 40% [1]. Pathogenesis of sepsis-induced ALI proceeds through an early phase characterized by granulocyte migration inside capillaries and monocyte extravasation, an intermediate phase of monocyte differentiation into macrophages (M*ϕ*s) inside alveoli, and a late phase of diffuse infiltration of alveoli by newly differentiated macrophages and extravasated neutrophils [2]. Recent studies have revealed that ALI is associated with enhanced apoptosis of pulmonary cells, including alveolar and airway epithelial cells as well as endothelial cells [3–5]. Inflammatory cell apoptosis is also enhanced in human with ARDS and in animal models of ALI [6].

The consequence of increased apoptosis in the lungs of septic patients is highly dependent on location of cellular apoptosis. While enhanced apoptosis of neutrophils and removal of apoptotic neutrophils by M*ϕ*s can attenuate the extent of tissue injury induced by neutrophils, lymphocyte apoptosis is detrimental to the septic host [7]. Likewise, exaggerated apoptosis of pulmonary epithelial and endothelial cells is harmful and leads to a worsening of gas exchange abnormalities. Yet despite the increasing importance of pulmonary apoptosis as a major contributor to the pathogenesis of ALI, factors that regulate pulmonary apoptosis in sepsis remain unclear. One possible initiator of pulmonary apoptosis is activated M*ϕ*s. In LPS-induced ALI models, monocyte migration into the lung vasculature and differentiation into M*ϕ*s precedes neutrophils infiltration [8]. M*ϕ*s play a pivotal role in the damage-repair process of the lung following sepsis-induced ALI [2]. They can modulate the neutrophil-mediated lung injury by engulfing apoptotic neutrophils [2] and secreting chemokines and chemoattractants that, in turn, induce neutrophil recruitment to the lung via increasing expression of cell adhesion molecules, such as ICAM-1 in endothelial cells and integrins in activated neutrophils. In addition, when activated, pulmonary M*ϕ*s produce relatively large amounts of proinflammatory cytokines and chemokines, including TNF-*α*, inteleukin-1, interleukin-8 and release reactive oxygen (ROS) and nitrogen (RNS) species derived from various enzymes, including NADPH oxidase and the inducible nitric oxide synthase [2, 9, 10]. Both ROS and RNS participate directly and indirectly in inducing tissue damage during the course of ALI. 

Recent studies have revealed that pre-treatment of rodents with gadolinium chloride (GdCl_3_, an inhibitor of Kupffer cells) prevents LPS-induced mortality, liver, and lung injuries and attenuates pro-inflammatory cytokine production and increased production of anti-inflammatory cytokines in these tissues [11–13]. The attenuation of ALI in endotoxemic animals pretreated with GdCl_3_ was attributed to a selective effect of GdCl_3_ on hepatic Kupffer cells, which are strongly inhibited and depleted by this compound, and to a reduction in pro-inflammatory cytokine production by pulmonary M*ϕ*s [12]. However, the possibility that reduced intensity of LPS-induced ALI in response to the inhibition of Kupffer cells by GdCl_3_ results in the attenuation of LPS-induced pulmonary apoptosis has never been addressed. In this study, we tested the hypothesis that blocking Kupffer cells with GdCl_3_ attenuates LPS-induced lung injury as a result of reduction in lung parenchymal apoptosis.

## 2. Methods

### 2.1. Experimental Design

The Animal Research Committee of McGill University approved all procedures. Pathogen-free male Sprague-Dawley rats (250–275 g) were housed in the animal facility of the hospital, were fed food and water *ad libitum*, and were studied 1 week after arrival. Four groups of animals were studied. Two groups (*n* = 9 in each group) were given an intraperitoneal injection of either normal saline (saline group) or *E. coli* lipopolysaccharide (serotype 055:B5, 15 mg/kg, LPS group, Sigma Inc., Oakville, ON, USA). Two additional groups (*n* = 9 in each group) of animals were pretreated with gadolinium chloride (7 mg/kg i.v., Sigma Inc.) 24 hrs prior to saline (saline+GdCl) or LPS (LPS+GdCl_3_) administration. All animals were anaesthetized with a mixture of ketamine, xylazine, and acepromazine and were killed by opening the thoracic cavity 12 hrs after saline or LPS injection. The lungs were then quickly dissected out, and one lobe was snap frozen in liquid nitrogen and stored at −80°C. The other lobe was used either for histological analysis or for measurement of lung water. For lung water assessment, lung lobes were weighed before (wet weight) and after drying overnight using a 50°C oven. Pulmonary water content index was calculated by subtracting the lung dry weight per body weight from the lung wet weight per body weight.

### 2.2. Lung Tissue Preparation and Immunoblotting

Frozen lung samples were homogenized with a metal homogenizer in 6 vol/wt ice-cooled homogenization buffer that contained (pH 7.5) 50 mM HEPES, 5 mM EDTA, 10% glycerol, 0.50% triton X-100, 1 mg/mL PMSF, 1 mM sodium orthovanadate, 5 *μ*g/mL aprotinin, 2 *μ*g/mL leupeptin, and 10 *μ*g/mL pepstatin A. The crude homogenates were centrifuged at 4°C for 30 min at 5,000 rpm, and the supernatants were used for immunoblotting experiments. Protein concentrations of homogenates were determined using the Bradford technique. Proteins (100 *μ*g) were loaded onto tris-glycine polyacrylamide gels and electrophoretically transferred onto PVDF membranes. For assessing the degree of pulmonary apoptosis, we detected the protein level of cleaved caspase-3 using a selective polyclonal antibody (New England Biolabs, Ipswich, MA, USA). Blots were scanned, and optical density of cleaved caspase-3 (17 kDa) was quantified using Image Pro Plus software (Media Cybernetics, Carlsbad, CA) and normalized as percentage of the mean optical density of the saline group detected on the same PVDF membrane. For assessment of pulmonary macrophage levels, we monitored the levels of ED1 macrophage antigen [14]. PVDF membranes were incubated with a monoclonal anti-ED1 antibody (Biosource International, Camarillo, CA, USA). Optical density of ED1 protein band (90–100 kDa) was quantified using scanning and ImagePro Plus software, as described above. 

### 2.3. Histology

One lung lobe was removed and placed in tissue-tek OCT (Sakura Finetek USA, Inc., Torrance, CA, USA) and perfused with a 1 : 1 mixture of tissue-tek OCT and phosphate buffer saline (PBS) via a tracheal catheter for 20 min at 25 cm H_2_O pressure. After inflation, the lungs were covered with tissue-tek OCT, immersed for 20 sec in cold isopentane, snap-frozen in liquid nitrogen, and stored at −80°C until sectioned by cryostat. For routine histology, sections (5 *μ*m thickness) were prepared with a cryostat, stained with hematoxylin and eosin (H&E), and covered with coverslips using permount. For neutrophil lung counting, sections were stained with a cytochemical stain that detects specific esterase enzymes (Sigma Inc.). Briefly, slides were fixed in citrate-acetone-formaldehyde solution for 30 sec, rinsed in deionized water and incubated with naphthol AS-D chloroacetate in the presence of freshly formed diazonium salt for 15 min at 37°C and protected from light. Slides were then rinsed thoroughly in deionized water for 2 min, counterstained in hematoxylin solution, rinsed in tap water, air dried, and covered with coverslips. Red cytoplasmic staining and lobulated blue nuclear staining identified neutrophils. Neutrophils were then counted in 30 randomly chosen fields (250x).

### 2.4. Myeloperoxidase Activity Assay(MPO)

 Myeloperoxidase activity, a general peroxidase activity, was performed as previously described [15]. Frozen lung samples were homogenized in 6 vol/wt ice-cooled homogenization buffer A (tris-maleate 10 mM, EGTA 3 mM, sucrose 275 mM, DDT 0.1 mM, leupeptin 2 *μ*g/mL, PMSF 100 *μ*g/mL, aprotinin 2 *μ*g/mL, pepstatin A 1 mg/100ml, pH 7.2). Samples were then centrifuged at 1000 g for 10 min. The pellet was discarded, whereas the supernatant was kept and designated as crude homogenate. Aliquots of this homogenate (50 *μ*g) were mixed with 1.4 ml of 50 mM phosphate buffer (pH 6.0) containing 0.167 mg/mL o-dianisidine dihydrochloride, and 0.0005% hydrogen peroxide. 200 *μ*L of the mixture was placed in a cuvette, and absorbance was measured at 460 nm every 2 min for a total of 30 min at 25°C. MPO activity was calculated as a change in absorbance/min/g protein.

### 2.5. Cytokine mRNA Expression

Total RNA was extracted from lung samples of five animals in each group using Trizol reagent, according to the manufacturer's instructions (Qiagen Inc., Valencia, CA, USA). The mRNA expression of different cytokines was measured by Multi-Probe RNase Protection Assay System (RPA) (RiboQuant by PharMingen, BD Biosciences, Mississauga, ON, USA). The MultiProbe Template Set allows the detection of IL-1-*α*, IL-1-*β*, TNF-*α*, TNF-*β*, IL-3, IL-4, IL-5, IL-6, IL-10, IL-2, and interferon (IFN)-*γ*. The housekeeping gene probes L32 and GADPH were included in the cytokine probe set for normalizing sampling and technique errors to permit comparison of individual mRNA species between samples, as was one positive control to ensure that the method was functioning correctly. Briefly, the multiprobe set was hybridized in excess to target RNA in solution, after which free-probe and other single-stranded RNA were digested with RNases. The remaining RNase-protected probes were purified, resolved on a denaturing polyacrylamide gel, and detected by autoradiography. The assay is specific and quantitative because of the sensitivity of RNase for mismatched base pairs and the use of solution-phase hybridization driven towards completion by excess probe. The intensity of mRNA expression was quantified by measuring the staining intensity of each band.

### 2.6. Apoptosis Measurements

Apoptosis was evaluated by immunoblotting for cleaved caspase-3, as described above. In addition, we quantified pulmonary apoptosis using a terminal deoxynucleotidyl transferase-mediated dUTP nick-end labeling assay (TUNEL). Lung sections were fixed with 4% paraformaldehyde and prepared according to manufacturer's instructions (In Situ Death Detection Kit, Roche Diagnostics Canada, Laval, QC). Slides were visualized with a light microscope. The staining was confirmed using both negative controls (TUNEL enzyme, TdT, was replaced with PBS) and positive controls (apoptosis was induced by DNase enzyme). For each animal, three-to-five slides were stained. In each slide, TUNEL-positive cells (identified by their purple-colored nuclei) were blindly counted in 30 randomly chosen (400x) fields. The mean value of these 3–5 slides per animal was reported and expressed as number of apoptotic cells/one 400 x fields.

### 2.7. Statistical Analysis

All results are expressed as means ± SE. One-way ANOVA test (SigmaStat, Jandel Scientific, San Rafael, CA, USA) was used for multiple comparisons. Statistical significance was set at *P* < 0.05.

## 3. Results

### 3.1. Lung Injury

**Figure 1:**
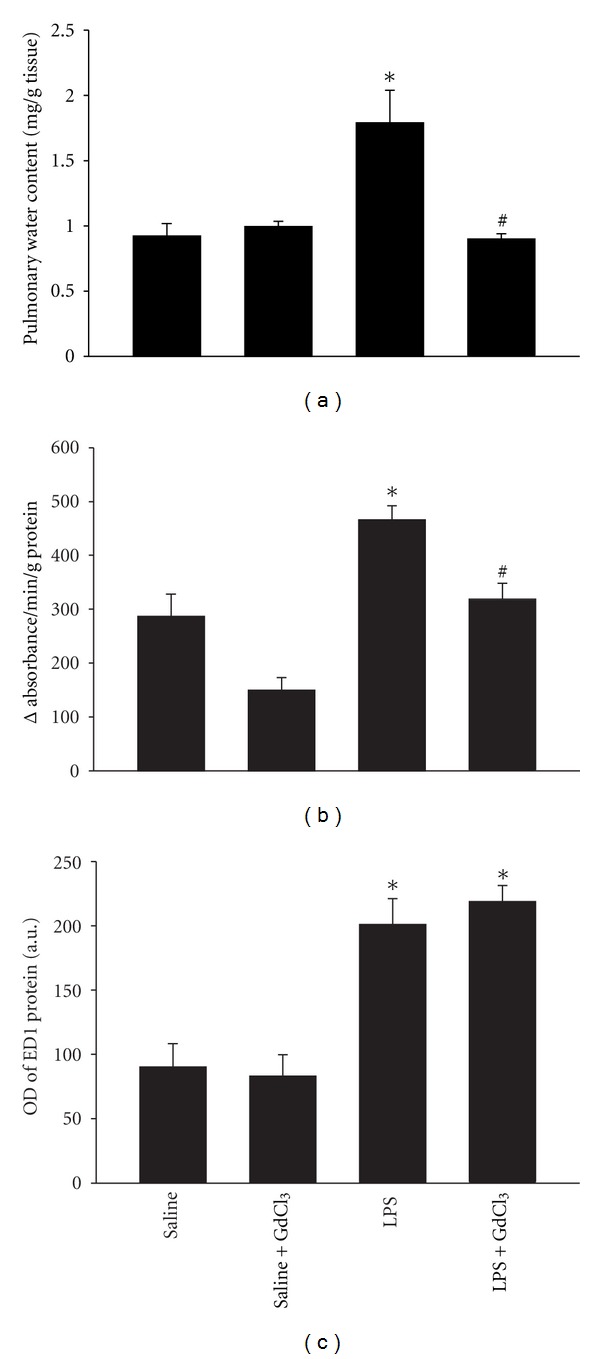
(a) Changes in pulmonary water content in the four groups of animals. *P < 0.05 compared with the saline group. ^#^ *P* < 0.05 compared with the LPS group. (b) MPO activity in lung homogenates in the four groups of animals. *P < 0.05 compared with the saline group. ^#^ *P* < 0.05 compared with the LPS group. (c) Mean ± SEM of optical densities of ED1 protein expression in the four groups of animals. *P < 0.05 compared with the saline group.

**Figure 2:**
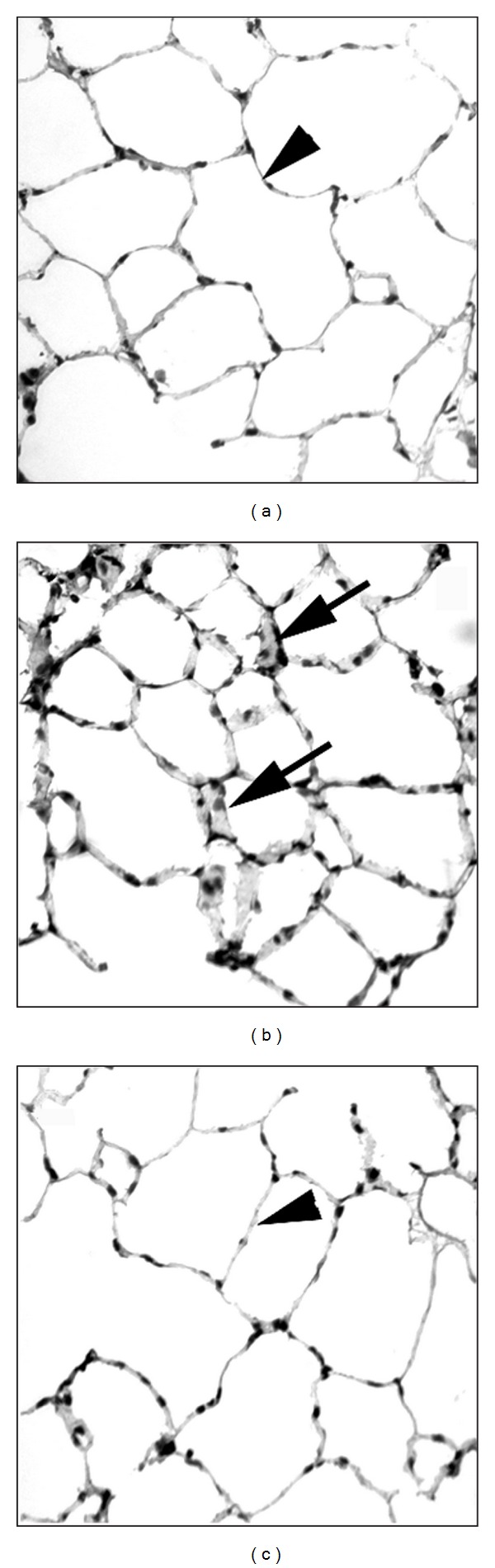
Hematoxylin and eosin-stained lung sections obtained from rats injected with saline (a), LPS (b), and LPS + GdCl_3_ (c). Full arrows indicate exudates in the alveolar spaces, whereas arrowheads indicate alveolar septa.

 Pulmonary water contents rose significantly in the LPS group compared with the saline group (Figure 1(a)). This rise in water content in response to LPS administration was attenuated by pretreatment with GdCl_3_ (Figure 1(a)). LPS administration had no influence on water content of the liver, heart, or kidney (data not shown). LPS administration induced a significant increase in lung MPO activity (*P* < 0.05, Figure 1(b)). Pretreatment with GdCl_3_ significantly attenuated lung MPO activity in the saline+GdCl_3_ and LPS+GdCl_3_ compared with the saline and LPS groups, respectively (*P* < 0.05, Figure 1(b)). Direct visualization and counting of neutrophils in lung sections showed a trend towards an increase in the LPS group (1.4 ± 0.01 cells/field) compared to the saline group (1.1 ± 0.2 cells/field). Pre-treatment with GdCl_3_ prior to LPS significantly decreased the number of lung neutrophils (0.9 ± 0.06 cells/field, *P* < 0.05 versus the LPS group). In comparison, a 9% decline in lung neutrophil count was observed in the saline+GdCl_3_ group compared with the saline group (not significant). LPS injection elicited a significant rise in pulmonary macrophage content compared with the saline group (Figure 1(c)). Pretreatment with GdCl_3_ had no influence on pulmonary macrophage content in the saline and LPS groups (Figure 1(c)). While no abnormalities were seen in the H&E staining of the lungs in the saline and saline+GdCl_3_ groups, thickening of alveolar septa, fluid exudation, and infiltration of inflammatory cells was observed in the lungs of the LPS group (Figure 2(b)). These changes were ameliorated by pretreatment with GdCl_3_ (Figure 2(c)).

### 3.2. Cytokine Gene Expression

**Figure 3:**
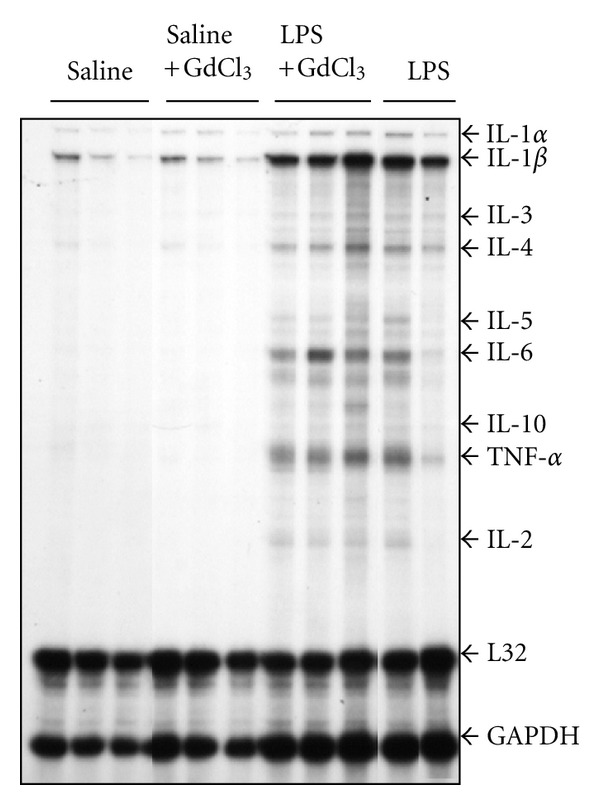
Representative examples of ribonuclease protection assays (RPA) showing mRNA expression of various cytokines.

**Figure 4:**
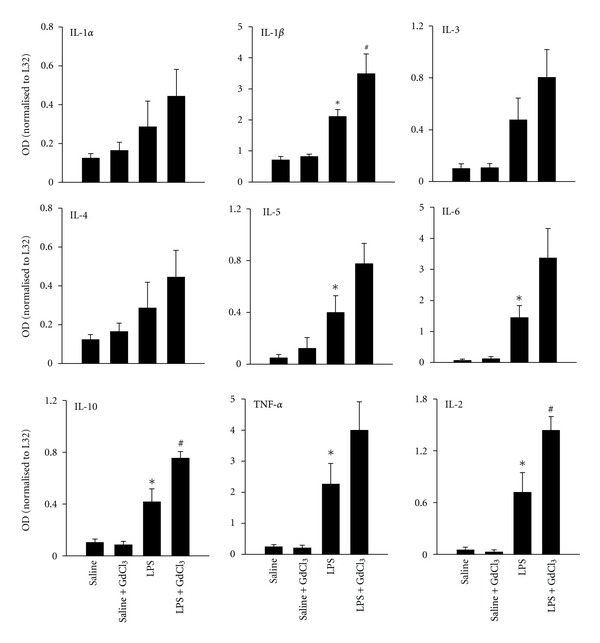
Mean ± SEM of optical densities of mRNA of various cytokines in the four groups of animals. *P < 0.05 compared with the saline group. ^#^ *P* < 0.05 compared with the LPS group.

Figure 3 illustrates a representative RPA assay for cytokine mRNA expression. In the saline and saline+GdCl_3_ groups, mRNA of IL-1*β* and, to a lesser extent, mRNA of IL-1*α* and IL-4 were detected. In comparison, mRNA of several other cytokines was also detected in the lungs of animals in the LPS and LPS+GdCl_3_ groups (Figure 3).  Figure 4 shows the mean optical densities of mRNA of various cytokines. IL-1*β*, IL-6, IL-5, IL-2, IL-10, and TNF-*α* mRNA expressions were significantly higher in the LPS group compared with the saline group (*P* < 0.05). Pre-treatment with GdCl_3_ did not change LPS-induced expression of most of these cytokines. However, a significantly greater increase in IL-1*β*, IL-10, and IL-2 mRNA expression was observed in the LPS+GdCl_3_ compared with the LPS group (*P* < 0.05 and < 0.01, Figure 4). 

### 3.3. Pulmonary Apoptosis

**Figure 5:**
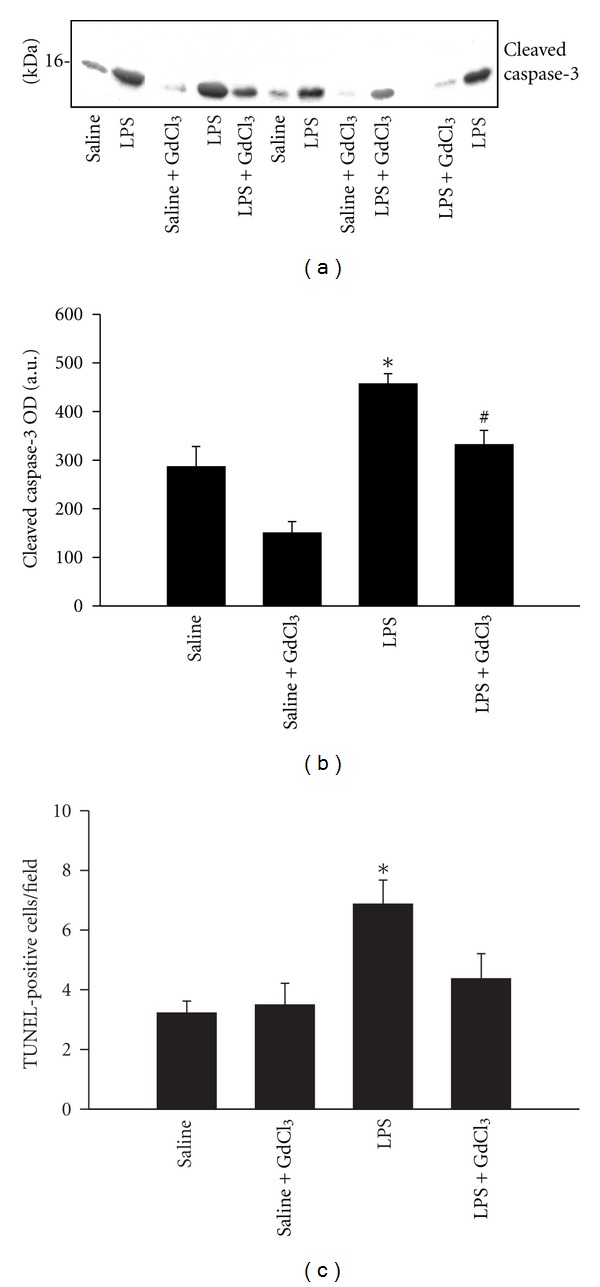
(a) and (b) Representative examples of cleaved caspase-3 and mean ± SEM of cleaved caspase-3 in lung lysates of the four groups of animals. *P < 0.05 compared with the saline group. ^##^ *P* < 0.05 compared with the LPS group. (c) Histogram showing the quantification of TUNEL-positive cells in the four experimental groups. TUNEL-positive cells were counted in 30 random fields and expressed as TUNEL-positive cells/HPF (400x). *P < 0.05 compared with the saline group. ^#^ *P* < 0.05 compared with the LPS group.

Relatively weak cleaved caspase-3 protein expression was observed in lungs treated with saline and saline+GdCl_3_ (Figure 5(a)). LPS administration triggered a significant rise in the intensity of cleaved caspase-3 activity. Pretreatment with GdCl_3_ in the LPS+GdCl_3_ group significantly reduced the intensity of cleaved caspase-3 (Figure 5(b)). Likewise, TUNEL staining revealed few positive cells in the lungs of saline and saline+GdCl_3_ groups. The majority of these cells appear to be located in the alveolar septa. LPS injection triggered a significant increase in the number of TUNEL positive cells compared with the saline group (Figure 5(c)). TUNEL-positive cells in LPS+GdCl_3_ group were reduced to about 65% of those found in the LPS group (Figure 5(c)). 

## 4. Discussion

The main findings of this study are (1) the injection of LPS in rats elicited acute lung injury as manifested by increased lung water content, neutrophil infiltration, pro-inflammatory cytokine gene expression and pulmonary apoptosis; (2) pretreatment with GdCl_3_ significantly attenuated the LPS-induced rise in pulmonary water content, neutrophil infiltration, and pulmonary apoptosis; (3) pre-treatment with GdCl_3_ had no influence on LPS-induced pulmonary pro-inflammatory gene expression, but it significantly increased the expression of the anti-inflammatory cytokine IL-10 as noticed in a previous report [12]. This cytokine was reported to decrease neutrophil recruitment and lung capillary leak in LPS-endotoxic mice [16]. This finding could explain the attenuation of neutrophil recruitment and pulmonary water content in LPS+GdCl_3_ group in our study. IL-10 was also reported to have an antipyretic effect in LPS-induced fever in mice [17].

GdCl_3_ is a lanthanide that is commonly used to evaluate the functional roles of liver macrophages in several processes including LPS-, ozone-, and hyperoxia-induced ALI [18–20]. While the exact mechanisms of action of GdCl_3_ are not yet clear, it has been proposed that it inhibits M*ϕ* phagocytosis by competitive blockade of K-type Ca^2+^ channels [21] and by reducing cellular expression of cytochrome P450s, thereby interfering with M*ϕ* metabolism [22]. Although we did not evaluate Kupffer cell inactivation in response to GdCl_3_ in the liver, GdCl_3_ is a very well-known and established inhibitor of Kupffer cells that was validated by many previous reports to inhibit Kupffer cell activation using a comparable or same dose of GdCl_3_ that we used in our study [12, 23–27]. 

Our results indicating that GdCl_3_ pre-treatment attenuated LPS-induced increase in pulmonary water contents, MPO activity, and histological indices of ALI are in agreement with previous studies [9, 12, 20]. Enhanced vascular leakage and increased edema formation in the lung of humans with ARDS, or animals with LPS-induced ALI, have been attributed to perturbed endothelial permeability brought about by several mediators, including increased ROS production and the release of proteases by activated neutrophils [20]. Administration of GdCl_3_ prior to intra-tracheal instillation of LPS strongly reduced pulmonary ROS production [20]. 

Kono et al. [12] have recently reported that pretreatment with GdCl_3_ attenuated the LPS-induced rise in the number of ED1- and ED3-positive cells in the liver and lungs. We have confirmed that the number of ED1-positive cells increased significantly in lungs of the LPS group compared with the saline group (Figure 1); however, GdCl_3_ pretreatment failed to alter this response. The reason behind these differences between our study and that of Kono et al. remains unknown; however, we speculate that differences in the concentrations of LPS and GdCl_3_ used in the two studies may be responsible. We used 7 mg/kg of GdCl_3_, followed by injection of 15 mg/kg LPS) while Kono et al. used 10 mg/kg of GdCl_3_, followed by 10 mg/kg LPS. *In vitro* studies have revealed that GdCl_3_ exerts a strong inhibitory effect on phagocytosis capability of Kupffer cells which is dose dependent and that only at relatively high concentrations did GdCl_3_ influence the viability of these cells [28]. 

We report here that cleaved caspase-3 intensity and the number of TUNEL-positive cells in the lungs rose significantly after 12 hrs of LPS administration (Figure 5). The choice of focusing on the 12 hrs time period post-LPS injection was based on our pilot experiments which revealed that cleaved caspase-3 intensity in the lungs peaked after 12 hrs of LPS administration with a decline thereafter. Similarly, Hamada et al. [24] confirmed that cleaved caspase-3 intensity peaked in the liver after 10 hrs of LPS administration. The functional implications of increased pulmonary apoptosis are dependent on the type of cells undergoing apoptosis. While increased apoptosis of alveolar epithelial and endothelial cells leads to major impairments of pulmonary gas exchange, apoptosis of inflammatory cells will likely cause a compromise in the adaptive immune responses and leave the lung susceptible to microorganism invasion. By comparison, increased apoptosis of neutrophils and, removal of these cells by M*ϕ*s will reduce the levels of pro-inflammatory cytokine release and the production of ROS and RNS derived from neutrophils, thereby attenuating the degree of tissue injury. We emphasize that our study reveals for the first time that pre-treatment with GdCl_3_ resulted in attenuation of LPS-induced pulmonary apoptosis. There are several possible mechanisms that could explain this observation. First, it is possible that pretreatment with GdCl_3_ might have attenuated the systemic responses to LPS by inhibiting the phagocytic properties of Kupffer cells thereby attenuating the cytokine cascade initiated by these cells leading eventually to blunting of local cytokine production by pulmonary parenchymal cells. This notion is supported by a recent report indicating that GdCl_3_ inhibits *in vitro* and *in vivo* LPS-induced activation of Kupffer cells, attenuates pro-inflammatory cytokine gene expression such as TNF-*α* and MIP-2, and increases gene expression of anti-inflammatory cytokine release including that of IL-10 and MCP-1 [12]. The attenuation of pro-inflammatory cytokine production by pulmonary parenchymal cells in response to LPS administration would likely result in reduction in apoptosis of these cells since pro-inflammatory cytokines including TNF-*α* are a known regulator of cellular apoptosis [29]. Although we did not measure Kupffer cell activation in our study, RPA measurements of total lung cytokine mRNA levels indicate that GdCl_3_ had no influence on LPS-induced pulmonary cytokine production (Figure 4). This finding suggests that pulmonary parenchymal cell responses to LPS administration remained intact in the presence of GdCl_3_ and argues against possible blunting of the systemic inflammatory response to LPS as a cause of reduced pulmonary apoptosis in animals treated with GdCl_3_. Second it is possible that GdCl_3_ might have influenced pulmonary apoptosis through its selective effect on phagocytic activity of pulmonary M*ϕ*s. Several reports have confirmed that GdCl_3_ inhibits pulmonary M*ϕ* activation in various models of ALI [9, 19, 30, 31]. More recently GdCl_3_ was reported to attenuate liver radiation-induced apoptosis in association with attenuated liver expression of TNF-*α*, IL-1*β*, and IL-6 [23]. Inactivation of Kupffer cells with GdCl_3_ attenuated LPS-induced liver apoptosis as estimated by caspase-3 activity and TUNEL- staining [24] in accordance with our results, and given that LPS caused disseminated endothelial apoptosis in lung, kidney, intestine and thymus [32] one can speculate that GdCl_3_ has a systemic anti-apoptotic effect in endotoxemic model.

The question which remains to be answered is how do activated pulmonary M*ϕ*s regulate apoptosis of lung parenchymal cells in response to LPS administration? One likely mechanism is through the activation of the Fas/FasL pathway. Fas receptors are cell death receptors, which are abundantly expressed in epithelial cells. Upon activation by Fas ligand (FasL), these receptors oligomerize and stimulate caspase-8, which triggers strong activation of the apoptosis machinery. Human monocytic cells (M*ϕ* precursors) contain high levels of Fas ligand [33] and when these cells are stimulated with an immune complex, Fas ligand rapidly translocates to the cell surface and is released as soluble FasL (sFasL) into the extra cellular milieu, thereby activating Fas of epithelial cells and inducing apoptosis in these cells. Activated M*ϕ*s also enhance the levels of sFasL by releasing metalloproteinase-9, which cleaves membrane-bound FasL and releases it into the intercellular space [34]. It is also possible that activated M*ϕ*s may induce pulmonary cell apoptosis indirectly through the release of chemoattractants that enhance neutrophil infiltration into the lung. These cells, in turn, release sFasL and induce epithelial and endothelial cell apoptosis [35]. Finally, activated M*ϕ*s release significant levels of ROS in the lungs of patients with ARDS and in animal models of ALI. ROS may directly induce apoptosis of pulmonary epithelial and endothelial cells [36, 37]. Thus, inactivation of pulmonary M*ϕ*s by GdCl_3_ and reduction of ROS derived from these cells will attenuate pulmonary apoptosis in LPS-injected animals. 

It should be emphasized that LPS can directly trigger cellular apoptosis through the activation of toll-like receptor 4 (TLR-4). Recently, Matsumura et al. [38] described a significant upregulation of TLR-4 mRNA in the lungs upon LPS administration in mice. TLR-4 receptors regulate cellular apoptosis through the pleiotropic transcription factor NF-*κ*B. In addition to activating TLR-4, LPS might also induce pulmonary cell apoptosis by directly mimicking ceramide, the second messenger of apoptotic pathways. Indeed, the lipid A moiety of LPS has structural similarities with ceramide and is capable of activating ceramide-activated protein kinase [39]. Our study does not rule out that a significant degree of LPS-induced apoptosis in the lung in response to LPS may be mediated through direct effects of LPS on pulmonary cells, and it is possible that the residual apoptosis detected in the lungs of the LPS+GdCl_3_ group could have been mediated through this mechanism. 

 In summary, we report here that pretreatment with GdCl_3_ resulted in significant reduction in LPS-induced vascular leakage, neutrophil infiltration, and pulmonary apoptosis, with no reduction in pro-inflammatory cytokine gene expression. These results suggest that GdCl_3_ attenuate sepsis-induced ALI mainly through the attenuation of pulmonary apoptosis rather than through reduction in pulmonary cytokine production.
